# The Role of Stereotypes in Jurors’ Indian Status Determinations and Guilt Decisions

**DOI:** 10.3390/bs15060824

**Published:** 2025-06-16

**Authors:** Kimberly Schweitzer, Dan Lewerenz

**Affiliations:** 1 Department of Psychology, University of North Dakota, 501 N. Columbia Road, Stop 8380, Grand Forks, ND 58202, USA; 2 School of Law, University of North Dakota, 215 Centennial Dr., Stop 9003, Grand Forks, ND 58202, USA; dan.lewerenz@und.edu

**Keywords:** Indian status, mock juror decision-making, defendant name stereotypicality, defendant photo stereotypicality

## Abstract

In the United States, courts say a jury must determine whether a defendant is an Indian subject to federal jurisdiction; however, jurors are provided little guidance regarding what to consider in their Indian status determinations. Given the jurisdictional and legal defense implications Indian status decisions have, we tested whether jurors consider two easily accessible potential indicators of Indian race: appearance and name. We examined whether mock jurors’ (*N* = 825) stereotypes of Indians influenced their determinations of whether a defendant is an Indian and whether that defendant is guilty of the crime alleged using a fully crossed 3 (defendant photo Indian stereotypicality: high, low, and none) × 3 (defendant name Indian stereotypicality: high, low, and none) between-participants design, controlling for participants’ feelings toward Indians as a group and internal and external motivations to respond without prejudice. In general, neither the defendant’s name nor photo stereotypicality predicted Indian status determinations, but jurors who thought the defendant was an Indian were more likely to find the defendant guilty. Thus, mock jurors consider factors other than the defendant’s name and appearance when deciding whether the defendant is Indian, but if the defendant is considered Indian, mock jurors are more likely to find the defendant guilty.

## 1. The Role of Stereotypes in Jurors’ Indian Status Determinations and Guilt Decisions

For nearly 140 years, the United States (U.S.) federal government has assumed responsibility for policing and prosecuting certain crimes committed involving Indians in Indian country. The Indian Major Crimes Act (1885) federalizes murder, kidnapping, arson, felony assault, and other selected “major” crimes when committed “within the Indian country” by an “Indian”.^1^ This exercise of federal jurisdiction is often explained as being necessary to protect Indians (*United States v. Kagama*, 1886). Although the U.S. Congress carefully crafted much of this framework, it left one critical element in the hands of juries: the determination of who is an Indian.

The U.S. Congress has defined “Indian” in dozens of statutes and regulations (e.g., Indian Reorganization Act, 1934; Indian Self-Determination and Education Assistance Act, 1975; Indian Arts and Crafts Act, 1990). However, the U.S. Congress has never defined “Indian” for federal criminal jurisdiction, leaving that question instead to the courts. Courts generally agree that to be an Indian subject to federal criminal jurisdiction, a person must have “some degree of Indian blood,” and be “recognized as an Indian by a tribe or the federal government or both” (*United States v. Stymiest*, 2009). The courts, in turn, delegate to juries the determination of whether a defendant is an Indian (*United States v. Stymiest*, 2009; *United States v. Zepeda*, 2015). To our knowledge, there is no empirical research examining how jurors determine whether a defendant is an Indian. This current research aims to fill this gap in the literature and provide insight into possible factors jurors may use to make legal Indian status determinations and examine how those Indian status determinations impact verdict decisions.

## 2. Historical and Legal Background

Historically, definitions of “Indian” have varied widely. Federally, who qualifies as an Indian is dependent on the purpose of the classification, as there are different requirements if the classification is for the census, the distribution of federal health and welfare benefits, or the recognition as a political entity (Beckenhauer, 2003). For example, the regulations governing how an unrecognized group of Indians may achieve federal recognition (Bureau of Indian Affairs, 2015) outline seven criteria to be a federally recognized tribe, including “…identified as an American Indian entity on a substantially continuous basis since 1900…” and “…membership consists of individuals who descend from a historical Indian tribe…”. To be eligible for most federal government services, individuals need to show membership in an Indian tribe that is federally recognized. Prior census data suggests these requirements are difficult to meet, as only approximately 50% of individuals who self-identified as Indian were eligible to receive federal benefits (Beckenhauer, 2003).

Tribal criteria for membership, however, vary by tribe, with some tribes requiring high proportions of Indian blood, others requiring mere lineal descendancy from some base roll, and still others imposing a residency requirement or some other requirement (Garroutte, 2001). The most common requirement for tribal citizenship is blood quantum, with approximately two-thirds of federally recognized tribes basing eligibility on specific blood quantum (Garroutte, 2001). The blood quantum required varies widely by tribe, with some tribes requiring 1/2 blood from their specific tribe (and some specifying further which parent it needs to be from) and others requiring 1/32 Indian blood, though 1/4 is the most common (Beckenhauer, 2003; Garroutte, 2001). Approximately 1/3 of tribes do not use specific blood quantum measurements to determine membership and instead require the person to be a direct descendant of another tribal member or an active participant in the Indian community and culture (Garroutte, 2001). This distinction between blood quantum-based and cultural-based definitions of Indian persists today and comes up in legal arguments when courts must determine Indian status (see Brown, 2020, for a discussion).

Jury instructions often state that jurors should not be influenced by the defendant’s race (e.g., Judicial Committee on Model Jury Instructions for the Eighth Circuit, 2023). In Indian status prosecutions, the court instructs jurors what factors they should consider when determining whether the defendant is an Indian (Judicial Committee on Model Jury Instructions for the Eighth Circuit, 2023; Ninth Circuit Jury Instructions Committee, 2022). Specifically, the jury must determine whether the defendant has “some degree of Indian blood” (although how much or how little Indian blood will suffice is not specified) and that the defendant “is recognized as an Indian person by a tribe or the federal government, or both” (Judicial Committee on Model Jury Instructions for the Eighth Circuit, 2023). In deciding that second factor, jurors are instructed that they may consider, among other factors, whether (1) the defendant is an enrolled member of a Tribe or Band, (2) a government recognizes the defendant as an Indian by providing assistance reserved only to Indians, (3) the defendant enjoys benefits of tribal affiliation, and/or (4) the defendant lives on a reservation or participates in Indian social life. These factors address both federal and tribal recognition, as well as the cultural components of being an Indian.

Although the jury is instructed both that they must determine that the defendant is an Indian to convict, and how to make that determination, the jury is not asked whether they determined that the defendant is an Indian. Rather, the jury (having previously been told that they cannot find the defendant guilty unless they determine that he is an Indian) is only asked whether the defendant is guilty or not guilty. In many Indian status cases, the defendant does not contest their Indian status. Where Indian status is contested, the government must put forth evidence of the defendant’s Indian status. Evidence in support of the first element (“some degree of Indian blood”) may come in the form of testimony (from tribal officials, federal government officials, or family members) or documentary evidence (tribal records, genealogical records, etc.). Evidence in support of the second element (“recognized as an Indian”) varies widely based on each defendant’s circumstances. Some examples of evidence in support of being recognized as an Indian include documentary or testimonial evidence that the defendant received federal government services and benefits provided to Indians (such as health care, education, etc.) and/or that the defendant received tribal services and benefits (such as health care, education, employment preference, hunting and fishing privileges, etc.); proof that the defendant resides or resided on an Indian reservation; or testimony that other Indians identified the defendant as an Indian.

Given the varying and inconsistent ways in which a person may meet these criteria, we propose that, in addition to the factors the courts instruct jurors to consider, jurors also consider other factors, including their assumptions based on stereotypes of what makes a person an “Indian” when determining Indian status, such as how they look or their name. Strictly speaking, the determination of Indian status is not a determination of the defendant’s “race”. The U.S. Supreme Court has held that, when the U.S. Congress legislates to carry out special legal obligations toward Indians, the U.S. Congress is not legislating on the basis of race, but rather on the basis of Indians’ unique political status (*Morton v. Mancari*, 1974). Specifically, the Court has held that, in subjecting Indians to federal criminal jurisdiction through the Major Crimes Act, Congress did not discriminate against Indians on the basis of race (*United States v. Antelope*, 1977). Nevertheless, as the current research demonstrates, questions of Indian status are inevitably intertwined with questions of race. Thus, the literature on race in the criminal legal system is relevant to our study of jury determinations of Indian status.

## 3. Perceptions of a Person’s Race and Stereotypicality

Research on how people determine another person’s race has found that the person’s appearance and name both impact their racial categorization. Social psychological and neuropsychological research finds that humans racially categorize others’ faces almost automatically (e.g., Rule & Sutherland, 2017), even when not asked to focus on the social demographic information of a person (e.g., Ito & Senholzi, 2013). The neurological relationship between event-related potentials (ERPs) and racial categorization also extends to later stereotypes and prejudice (see Amodio et al., 2021 for a review). For example, the magnitude of racial bias in participants performing the shoot, no-shoot task (where participants are told to “shoot” anyone they see on the screen holding a gun and not “shoot” anyone holding a different object) was positively correlated with the same ERPs tied to racial categorization (Correll et al., 2006). In other words, neurological evidence of racial categorization is tied to later prejudicial actions (e.g., being quicker to “shoot” a Black person holding a gun compared to a White person). Further, individuals with more stereotypical facial features of a particular race are more likely to be negatively evaluated and thus subjected to negative stereotypes, prejudice, and discrimination (e.g., Maddox, 2004).

Given that Native American is often chosen as an identity of multi-racial individuals (Ghoshal, 2024; Parker et al., 2015), one area of research that is particularly informative to the current work is research examining perceptions of multi-racial or racially ambiguous individuals (see Pauker et al., 2018, for a review and Young et al., 2021, for a meta-analysis). In general, research finds that less distinct racial presentations (e.g., Indian) are harder to categorize (e.g., Chen & Hamilton, 2012; Freeman et al., 2010), and racial categorization can be dependent on the context and specific circumstances of the categorization (e.g., Chen et al., 2014; Freeman et al., 2011). Further, an individual’s own biases and prejudices can lead to categorizations of mixed-race or racially ambiguous people as the race the perceiver has a more negative bias toward (Ho et al., 2015). In their study, Ho et al. (2015) found that mixed-race targets were more likely to be categorized as Black instead of White if the perceiver had a negative bias against Black individuals and believed in racial essentialism. Feliciano (2016) also found people tended to racially categorize mixed-race individuals into one racial category and found support for a third mono-racial category of Latino (in addition to White and Black). In general, people categorize faces by skin color, with lighter-skinned individuals being classified as White, medium-skinned as Latino, and darker-skinned as Black (Feliciano, 2016). Given there are several other racial categories, the question becomes what other factors individuals use to categorize people based on race.

A second factor shown to affect perceptions of race is the name of the target. Garcia and Abascal (2016) presented participants with a racially ambiguous photo and manipulated the name of the person to be either stereotypically Hispanic or non-Hispanic. When the name was stereotypically Hispanic, participants rated the skin tone of the photo as darker than when the name was stereotypically non-Hispanic. This effect was amplified when the photo was of a male (Garcia & Abascal, 2016). This research suggests that the racial stereotypicality of a name can alter perceptions of the race of a person in a photograph, as can the gender of the person in the photo (but not the perceiver).

Schachter et al. (2021) expanded on the prior work reported and examined not just skin tone and name, or just White, Black, and Hispanic, but also ancestry and language spoken, as well as Native American, Asian, and Middle Eastern/North African groups. Focusing on the Native American findings, Schachter et al. (2021) found ancestry (i.e., Cherokee/Navajo) was the strongest predictor of participants classifying the target person as Native American, followed by language spoken (i.e., Navajo), skin tone (i.e., medium), and name (i.e., Odakota or Lakota). These findings were replicated by Ghoshal (2024) who found that ancestry was the number one factor when deciding a Native American person’s race (as well as White and Black, but not Hispanic). The second most important criterion for determining race for Native American individuals was whether they met the criteria for membership established by a federally recognized tribe. Whether their family lived on a reservation and their cultural upbringing were ranked seven and eight, respectively. When factoring in the racial identity of the rater, multi-racial raters who identified as partially Native American rated genetics and ancestry as more important than White and Black raters (Ghoshal, 2024).

In sum, there are multiple different indicators people use to determine another person’s race, and the importance of the indicator is race-specific. The little research that has examined different racial indicators of Native American or Indian individuals finds that skin color can be used, but the person’s name is a more reliable indicator, along with ancestry, meeting federal criteria for tribal membership, and language spoken (Ghoshal, 2024; Schachter et al., 2021). The current research chose to focus on name and appearance to examine how these factors affect legal decisions regarding the Indian status of a defendant as they are both factors that are always available to jurors.

## 4. Defendant Race and Jurors’ Decisions

The impact of a defendant’s race on jurors’ decisions has received relatively substantial attention from psycho–legal scholars. That said, the research is far from consistent or conclusive, with some research finding little to no overall effect of defendant race on verdicts (e.g., Devine & Caughlin, 2014). The majority of the defendant race scholarship has examined differences between White and Black defendants, while some research has also examined Hispanic defendants. Generally speaking, Black defendants are more likely to be found guilty by jurors (Mitchell et al., 2005; see Devine & Caughlin, 2014, for nuances). This effect is amplified if the juror is White, as research has found jurors tend to have an in-group bias (e.g., Leippe et al., 2021). White mock jurors tend to be more punitive when the defendant is Black compared to White, and Black mock jurors tend to be more punitive when the defendant is White compared to Black (Sommers & Ellsworth, 2000, 2001). Another study found the biggest bias was with White mock jurors and Hispanic defendants (compared to White defendants), with Hispanic defendants receiving more severe judgments (Devine & Caughlin, 2014). Adding to the nuance, the race of the victim has also been shown to impact the effect of the defendant’s race on jurors’ decisions. ForsterLee et al. (2006) found jurors were least punitive when both the defendant and victim were White and most punitive when the defendant was White and the victim was Black.

Much less research has examined the effect of an Indian defendant on jurors’ decisions, but like the White/Black defendant literature, the findings are mixed and nuanced. Regarding guilt determinations, some research finds no effect of defendant race on verdict (Ewanation & Maeder, 2018; Maeder et al., 2015b, 2015a, Study 1; Struckman-Johnson et al., 2008; Willis Esqueda & Swanson, 1997) or sentencing (Willis Esqueda & Swanson, 1997), while other research finds Indian or Canadian Aboriginal defendants are more likely to be found guilty (Maeder & Burdett, 2013; Maeder et al., 2015a, Study 2) and receive harsher sentences (Hall & Simkus, 1975; Maeder et al., 2015b) compared to White defendants. Within this research, race was manipulated by stating the defendant’s race, altering the defendant’s name to be stereotypical of the different races, and/or altering the defendant’s photo to be stereotypical of the different races. These different methods of manipulation may partially explain the discrepant findings. Further, given the confounding nature of manipulating both the defendant’s name and photo simultaneously, we are unable to tease apart if it is the defendant’s name or photo that can lead to biased decisions by jurors. As such, the current study sought to test how the stereotypicality of the defendant’s name and photo, specifically for Indian defendants, may alter verdict decisions by manipulating both the stereotypicality of the defendant’s name and the stereotypicality of the defendant’s photo in a fully crossed between-participants design.

A second inconsistency in the previous literature examining the effect of a defendant being Indian is whether or not the participant’s own racial biases were controlled for. As Ewanation and Maeder (2018) found, participants believed Indigenous people (compared to Whites) were more likely to be stereotyped as criminals, and participants who felt this were less likely to find the defendant guilty. Although scholars have used a variety of scales and measures to assess mock juror racial bias, it is clear that racial bias is an important factor to consider and control for when testing possible defendant race effects on trial outcomes. Additionally, the bulk of the literature examining how a defendant being Indian affects verdicts and sentencing has used the context of Canadian societal groups and the Canadian legal system. Given the somewhat different history and experiences of Indians in the United States (compared to Canadian Aboriginal individuals) and the different legal jurisdictional questions being an Indian raises in the U.S., we felt it was important to examine the effect of the defendant being Indian on U.S. mock jurors’ decisions.

## 5. The Current Research

The goal of the current research was to examine the influence of the stereotypicality of a defendant’s name and photo on mock jurors’ perceptions of whether the defendant can be legally classified as an Indian and whether the defendant is guilty of the alleged crime in a U.S. criminal case context. This research is critically important if Indians are to receive fair trials in federal courts. The existing scholarship criticizes the composition of juries in Indian status cases (e.g., Gross, 2016; Washburn, 2008) and suggests that whether a defendant is an Indian can sometimes bias verdicts and sentencing; but to date, no one has sought to empirically test how juries make Indian status determinations. Given Schachter et al.’s (2021) findings that name and skin tone both increased categorization to Native American by two points and Garcia and Abascal’s (2016) findings suggesting the racial stereotypicality of a name can alter perceptions of the race of a person in a photograph, we sought to test the interactive effect of a defendant’s name and photo on mock jurors’ decisions. Based on the existing literature reviewed, we propose the following hypotheses to address our two main research questions: (1) do mock jurors come to different determinations of Indian status based on the stereotypicality of the defendant, and (2) does a mock juror’s decision regarding the Indian status of the defendant impact juror verdict decisions.

### 5.1. Main Effects of the Stereotypicality of the Defendant’s Name and Photo

In line with research showing that skin tone and name predicted whether participants classified the target person as Native American (Schachter et al., 2021), we predicted main effects of the stereotypicality of the defendant’s name and photo. When the defendant’s name or photo is more stereotypically Indian, participants will be more likely to say the defendant has some degree of Indian blood, is recognized as an Indian person by a tribe or the federal government, is an Indian, and is guilty of assault compared to when the name or photo is less stereotypically Indian or not provided.

### 5.2. Interactive Effects of the Stereotypicality of the Defendant’s Name and Photo

In line with research showing that the racial stereotypicality of a name can alter perceptions of the race of a person in a photograph (Garcia & Abascal, 2016), we predicted an interactive effect of the defendant’s name and photo and perceptions of Indian Status. When the defendant’s name and photo are both highly stereotypically Indian, participants will be most likely to say the defendant has some degree of Indian blood, is recognized as an Indian person by a tribe or the federal government, is an Indian, and is guilty of assault, compared to when the defendant’s name and photo are not provided or are less stereotypically Indian.

### 5.3. Main Effects of Participants’ Feelings Towards Indians as a Group and Internal and External Motivations to Respond Without Prejudice

In line with prior research regarding general racial prejudice by jurors (e.g., Ewanation & Maeder, 2018) and the effect of racial biases on perceptions of race (e.g., Ho et al., 2015), we predicted that participants who report lower feelings of warmth toward Indians as a group and have lower internal and external motivations to respond without prejudice to Indians will be more likely to say the defendant has some degree of Indian blood, is recognized as an Indian person by a tribe or the federal government, is an Indian, and is guilty of assault compared to participants that report greater warmth toward Indians and have higher internal and external motivations to respond without prejudice.

### 5.4. Main Effects of Participant’s Indian Status-Related Decisions

Given the mixed findings regarding Indian race defendants and guilt decisions (e.g., Maeder & Burdett, 2013; Maeder et al., 2015b; Willis Esqueda & Swanson, 1997), we did not propose a priori hypotheses regarding how mock jurors’ decisions regarding the Indian status of a defendant may affect their verdict decisions.

## 6. Method

### 6.1. Participants and Design

Participants (*N* = 954) were recruited via Prolific, an online participant recruitment platform where individuals sign up to complete tasks for money. Power analyses indicated that, to be sufficiently powered (power = 0.8) for multivariate logistic regression models with estimated probabilities of 0.45 (p_0_) and 0.50 (p_1_), 811 participants were necessary. After removing participants who were not jury-eligible in most states (i.e., non-US citizen and/or history of a felony conviction, *n* = 57) and who failed one or more attention check questions (*n* = 77), 825 participants remained in our sample. Of these participants, the average age was 41.11 (*SD* = 12.78), 55.27% identified as male, 42.91% identified as female, 1.82% identified as non-binary or other, 71.39% identified as White, 13.82% as Black, 6.91% as Asian, 7.63% as mixed or other, and 0.24% as American Indian or Alaska Native.

The design of the current study was a 3 (defendant name stereotypicality: none, low, or high) × 3 (defendant photo stereotypicality: none, low, or high) between-participants design. Our dependent variables were participants’ decisions regarding the defendant’s Indian status and guilt.

### 6.2. Materials and Procedure

All study materials were approved by our university’s institutional review board and presented to participants online via Qualtrics, a survey creation tool. Study materials can be found on our project’s Open Science Framework (OSF) page at https://osf.io/6w5gj/?view_only=55149050aa9440beaf9e69df98245c8e (accessed on 15 March 2025). 

### 6.3. Pilot Study

To ensure the defendant’s name and photo manipulations varied in how stereotypical Indian people perceived them to be, we first conducted a pilot study. As in the main study, participants (*N* = 103) were recruited from Prolific. After removing participants who failed one or more attention check questions (*n* = 28), 75 participants remained in our sample. Of these participants, the average age was 36.64 (*SD* = 12.46), 36.00% identified as male, 48.00% identified as female, 66.67% identified as White, 6.67% as Black, 14.67% as Asian, 12.00% as mixed or other, and 0% as American Indian or Alaska Native.

Participants were randomly presented with 10 different names and 10 different photos and were asked to respond quickly to provide their gut reaction to each name and photo. Names were derived from the Eleventh U.S. Census (United States Census Bureau, 1890)—Volume 10: Report on Indians Taxed and Not Taxed in the United States (Except Alaska). Photos were found online and were mugshots of men charged as Indians under the Indian Major Crimes Act (see the OSF for the 10 photos). Thus, all names and photos used for the pilot represented names and photos of Indians. For each name and photo, participants were asked to rate how closely the name/photo matched their idea of an American Indian or Alaska Native individual on a 0–100 sliding scale. To ensure the names chosen for the main study did not significantly differ on other characteristics that could influence trial outcomes, we also asked participants to rate or guess the person’s age, attractiveness, social class, and likelihood of a criminal history. The study took participants approximately 10 min to complete, and all participants were paid USD 1.75.

Two names were rated as highly stereotypical of Indians (Tall Chief [*M* = 83.11, *SD* = 22.99] and Black Coyote [*M* = 80.55, *SD* = 25.29]), and three names were rated low in Indian stereotypicality (David Faulkner [*M* = 10.48, *SD* = 15.60], George McDaniel [*M* = 12.00, *SD* = 15.50], and Will P. Thompson [*M* = 12.59, *SD* = 16.35]). The name Tall Chief was rated as significantly older than all other names (all *p*s < 0.001), but there were no other significant differences in perceived age between the other names. There were no significant differences between any of the five names on attractiveness; Tall Chief was rated as the most attractive of the five names, with the largest difference being between Tall Chief and George McDaniel (*p* = 0.09). The two highly stereotypical names were rated as being of significantly lower social class than all three of the less stereotypical names (all *p*s < 0.001), with Black Coyote rated as having the lowest social class. Regarding the perceived likelihood of criminal history, Tall Chief was rated as least likely to have a criminal history, and Black Coyote as most likely (*p* < 0.001). The only other significant difference was between Black Coyote and David Faulkner (*p* = 0.045). Considering all this information, we chose George McDaniel as the less stereotypically Indian name and Black Coyote as the more stereotypically Indian name.

Similar analyses were conducted with the photos, with two rated as highly stereotypical of Indians (Photo 10 *M* = 75.05, *SD* = 22.03; Photo 8 *M* = 73.04, *SD* = 20.23) and three rated as less stereotypically Indian (Photo 5 *M* = 14.33, *SD* = 19.40; Photo 9 *M* = 15.61, *SD* = 19.41; Photo 4 *M* = 16.33, *SD* = 18.98). After comparing these photos on perceived age, attractiveness, social class, and the likelihood of having a criminal history, Photo 9 was chosen as the less stereotypically Indian photo, and Photo 10 as the more stereotypical Indian photo. See Table 1 and Table 2 on the OSF page for descriptive statistics from the pilot study.

### 6.4. Main Study

After consenting to participate, participants received a trial summary transcript and audio recording containing basic facts of the case that lasted approximately 27 minutes. The trial summary was written by an attorney who specializes in Indian law and was based on prior real-life cases. Participants were told the defendant was on trial for simple assault. The trial summary contained (1) a closing argument from the prosecution summarizing evidence indicative of guilt for each element of the offense, (2) a closing argument from the defense summarizing evidence against guilt for each element of the offense, and (3) jury instructions from the judge instructing jurors generally on the duty of the jury and specifically on the elements of the offense (outlined below as numbers 1–3) and how they were to determine each element.

In four of the six conditions, along with the trial summary, one of two photos of the defendant appeared at the top of the page. The photo depicted an individual rated in the pilot study to be highly stereotypical of an Indian in the high stereotypicality photo condition or one rated in the pilot study to be low in Indian stereotypicality in the low stereotypicality photo condition. The defendant’s photo remained at the top of the page the entire time participants listened to the trial summary (approximately 27 min).

The name of the defendant was manipulated throughout the closing arguments by both sides. In the condition with no indication of stereotypicality, the defendant was simply referred to as “the defendant”. Using the results from the pilot study, in the highly stereotypical name conditions, the defendant was referred to as Black Coyote or Mr. Coyote, and in the low stereotypicality name conditions, the defendant was referred to as George McDaniel or Mr. McDaniel. The defendant’s name appeared 61 times throughout the closing arguments.

After listening to and reading the trial summary, all participants then answered whether they believed the prosecution proved, beyond a reasonable doubt, each of the elements (1–3) and sub-elements (3a and 3b) of the crime alleged:

(1) The defendant assaulted the victim;
(2) The assault happened on the Indian Reservation;
  (3a) The defendant has some degree of Indian blood;
  (3b) The defendant is recognized as an Indian person by a tribe or the federal government, thus
(3) the defendant is an Indian.

Participants then responded to whether they believed the prosecution proved, beyond a reasonable doubt, that the defendant was guilty of assault by an Indian on an Indian reservation, which served as their formal guilty/not guilty decision. Following the post-trial questions, participants completed three multiple choice questions asking basic information about the trial (e.g., what was the defendant on trial for) to ensure they were paying attention and one question directly assessing the manipulation (i.e., what was the race of the defendant).

Next, participants rated the degree of warmth they felt for different racial categories (as well as other unrelated categories) using a 7-point Likert scale (1 = *feel least warm towards this group*, 4 = *neutral*, 7 = *feel most warm towards this group*). The categories (American Indian or Alaskan Native, White, Black or African American, Hispanic/Latinx, Asian, Native Hawaiian or Other Pacific Islander, elderly, young, disabled, poor, and rich) were presented in a random order to participants.

Participants then completed an adapted version of the internal and external motivation to respond without prejudice scale (IMS/EMS; Plant & Devine, 1998). Instead of saying “black people” in each item, this was replaced with “American Indians”. For example, participants responded to, “I try to act non-prejudiced toward American Indians because of pressure from others”. adapted from the original, “I try to act nonprejudiced toward Black people because of pressure from others”. Ten 9-point Likert questions assessed IMS and EMS (five questions each). The adapted scale showed good reliability in the present study (IMS α = 0.86, EMS α = 0.86).

Last, participants provided their age, sex, race, whether they are U.S. citizens (required to serve on a U.S. jury), and whether they have ever been convicted of a felony (which would exclude them from jury service in many jurisdictions). The study took approximately 37 min to complete and, upon completion, participants were debriefed and given a code to receive compensation (USD 7.00).

## 7. Results

All analyses were performed using the statistical program R (R Core Team, 2024) and its base packages. The following additional packages were used to restructure and examine the data, probe interactions, and create tables: dplyr (Wickham et al., 2023), psych (Revelle, 2024), effects (Fox & Weisberg, 2019), and sjPlot (Lüdecke, 2024).

To answer our first research question—Do mock jurors come to different determinations of Indian status based on the stereotypicality of the defendant?—we ran four hierarchical multivariate logistic regression models. In the first step of each model, the main effects of the stereotypicality of the defendant’s name and photo were entered as predictors with the highly stereotypical name and photo being the reference categories to allow for a comparison between low and high stereotypicality. In the second step of each model, the interactions between name and photo were entered as predictors, and in the third step of each model, participants’ feeling thermometer ratings for Indians and IMS and EMS scores were entered as predictors.

**Figure 1:**
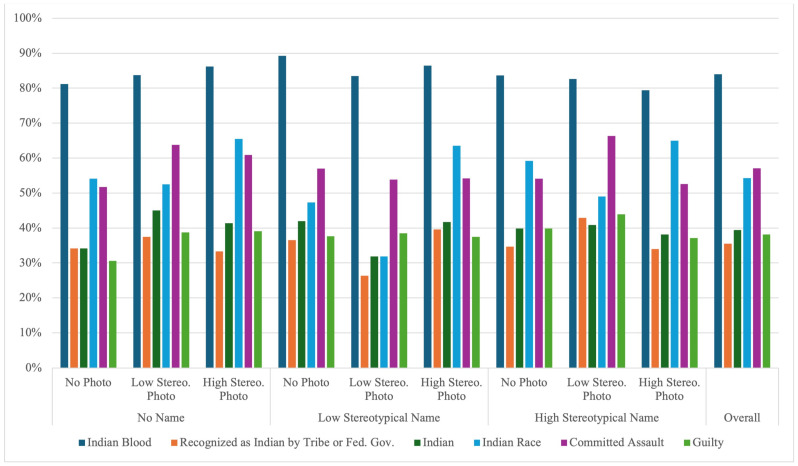
The Percentage of Participants Who Said Yes Given the Defendant’s Name and Photo Condition.

Three dependent variables addressed the legal determinations of Indian status: whether the defendant (1) has some degree of Indian blood; (2) is recognized as an Indian person by a tribe or the federal government, or both; and (3) is an Indian. Along with the Indian status variables, we also explored predictors of participants’ racial categorization of the defendant in a fourth, exploratory regression analysis. The racial categorization variable was presented after the trial decisions (including guilt) and simply asked participants what race they thought the defendant was from a standard list of racial categories (i.e., Asian, American Indian or Alaska Native, Black or African American, Native Hawaiian or Other Pacific Islander, White, Other, and Unsure), which we then categorized as either Indian (American Indian or Alaskan Native; coded as 1) or not Indian (coded as 0). See Figure 1 for descriptive statistics.

### 7.1. Degree of Indian Blood

Regarding the model for whether participants thought the defendant had some degree of Indian blood (*r*^2^ = 0.02), neither the stereotypicality of the defendant’s name nor photo (nor the interactions between the two) significantly predicted the participants’ decisions. Participants’ feeling thermometer ratings for Indians and their internal motivations to respond without prejudice also did not significantly predict Indian blood determinations. Participants with higher external motivations to respond without prejudice towards Indians were significantly less likely to think the defendant had some degree of Indian blood compared to participants with lower external motivations to respond without prejudice (β = −0.11, *SE* = 0.05, *p* = 0.02). See Table 1 for full model statistics.

**Table 1:** Hierarchical multivariate logistic regression model with name and photo stereotypicality, feeling thermometer, and internal and external motivations to respond without prejudice predicting Indian blood determinations (0 = No, 1 = Yes).

	Step 1			Step 2			Step 3		
*Predictors*	*Odds Ratios*	*CI*	*p*	*Odds Ratios*	*CI*	*p*	*Odds Ratios*	*CI*	*p*
Intercept	4.50	3.06–6.77	**<0.001**	3.85	2.40–6.47	**<0.001**	3.76	1.18–12.62	**0.03**
Low Stereotypical Name	1.41	0.90–2.22	0.14	1.66	0.78–3.63	0.19	1.79	0.84–3.94	0.14
No Name	1.14	0.73–1.78	0.58	1.62	0.75–3.64	0.23	1.80	0.82–4.06	0.15
Low Stereotypical Photo	0.96	0.61–1.51	0.85	1.24	0.60–2.56	0.56	1.29	0.63–2.69	0.49
No Photo	1.07	0.68–1.70	0.77	1.33	0.64–2.79	0.44	1.41	0.68–2.96	0.36
Low Stereo. Name × Low Stereo. Photo				0.64	0.22–1.88	0.42	0.62	0.21–1.83	0.39
No Name × Low Stereo. Photo				0.67	0.22–2.03	0.48	0.59	0.19–1.80	0.35
Low Stereo. Name × No Photo				0.98	0.31–3.09	0.97	0.92	0.29–2.91	0.88
No Name × No Photo				0.52	0.17–1.54	0.24	0.48	0.16–1.44	0.19
Feeling Thermometer							1.04	0.88–1.24	0.62
IMS							1.02	0.90–1.15	0.74
EMS							0.89	0.82–0.98	**0.02**
*N*	825			825			825		
*R*^2^ Tjur	0.003			0.006			0.02		

Note. Bolded *p*-values indicate *p* < 0.05. For the name and photo stereotypicality predictors, the high stereotypicality condition is the reference category.

### 7.2. Recognized by Tribe or Federal Government

When predicting whether participants thought the defendant was recognized by the federal government as an Indian (*r*^2^ = 0.02), all predictors were non-significant except the interaction between the low stereotypical name and low stereotypical photo (β = −0.98, *SE* = 0.44, *p* = 0.02) and IMS (β = −0.10, *SE* = 0.05, *p* = 0.048). When the defendant’s name and photo were less stereotypically Indian, participants were significantly less likely to say the defendant was recognized by the federal government as an Indian (26%) compared to when the defendant’s photo was less stereotypically Indian and the name was highly stereotypical (42%, χ^2^ (1, *n* = 189) = 4.93, *p* = 0.03). No other comparisons were statistically significant. Participants with higher internal motivations to respond without prejudice towards Indians were significantly less likely to think the defendant had some degree of Indian blood compared to participants with lower internal motivations to respond without prejudice. See Table 2 for full model statistics.

**Table 2:** Hierarchical multivariate logistic regression model with name and photo stereotypicality, feeling thermometer, and internal and external motivations to respond without prejudice predicting recognition by federal government as Indian determinations (0 = No, 1 = Yes).

	Step 1			Step 2			Step 3		
*Predictors*	*Odds Ratios*	*CI*	*p*	*Odds Ratios*	*CI*	*p*	*Odds Ratios*	*CI*	*p*
Intercept	0.60	0.44–0.81	<0.01	0.52	0.34–0.78	<0.01	0.59	0.23–1.49	0.26
Low Stereotypical Name	0.88	0.63–1.24	0.47	1.27	0.71–2.29	0.42	1.26	0.70–2.28	0.44
No Name	0.91	0.64–1.29	0.58	0.97	0.52–1.79	0.92	0.92	0.50–1.71	0.80
Low Stereotypical Photo	1.00	0.70–1.41	0.99	1.45	0.82–2.61	0.21	1.40	0.78–2.52	0.26
No Photo	0.97	0.69–1.38	0.88	1.03	0.57–1.86	0.92	1.01	0.56–1.84	0.96
Low Stereo. Name × Low Stereo. Photo				0.38	0.16–0.88	**0.02**	0.38	0.16–0.88	**0.02**
No Name × Low Stereo. Photo				0.82	0.35–1.95	0.66	0.86	0.36–2.05	0.73
Low Stereo. Name × No Photo				0.85	0.37–1.97	0.71	0.86	0.37–1.98	0.71
No Name × No Photo				1.01	0.42–2.39	0.99	1.04	0.43–2.48	0.94
Feeling Thermometer							1.10	0.96–1.26	0.15
IMS							0.91	0.83–1.00	**0.05**
EMS							1.04	0.97–1.12	0.29
*N*	825			825			825		
*R*^2^ Tjur	0.001			0.008			0.02		

Note. Bolded *p*-values indicate *p* < 0.05. For the name and photo stereotypicality predictors, the high stereotypicality condition is the reference category.

### 7.3. Indian Status

None of our variables significantly predicted participants’ Indian status determinations (*r*^2^ = 0.01; see Table 3 for model statistics). Of note, seven participants who believed the defendant did not have some degree of Indian blood also believed the defendant was an Indian. Similarly, 50 participants who did not believe the defendant was recognized as an Indian by a tribe or the federal government also believed the defendant was an Indian.

### 7.4. Indian Race

We also explored whether participants’ perceptions of the defendant’s race were altered by our name and photo manipulations. When predicting whether participants categorized the defendant’s race as Indian (*r*^2^ = 0.04), photo stereotypicality was the only significant predictor. When the defendant’s photo was more stereotypically Indian, participants were significantly more likely to say the defendant was of Indian race (40%) compared to when the defendant’s photo was less stereotypically Indian (26.6%, β = 0.66, *SE* = 0.29, *p* = 0.03). See Table 4 for full model statistics.

### 7.5. Verdict

To answer our second research question—Does a mock juror’s decision regarding the Indian status of the defendant impact juror verdict decisions?—we ran a hierarchical logistic regression model (*r*^2^ = 0.80). In the first step, we entered two factors that jurors were instructed to consider in their guilt decisions: the defendant’s Indian status and whether the defendant assaulted the victim. In the second step, we added the additional Indian status questions used as dependent variables in the earlier models: whether the defendant (1) had some degree of Indian blood; (2) was recognized as an Indian person by a tribe or the federal government, or both; (3) is an Indian; and (4) is racially categorized as an Indian. In the third step, we added the feeling thermometer and IMS/EMS measures.

Whether the defendant was an Indian, assaulted the victim, was recognized by a tribe or the federal government, or was categorized racially as an Indian consistently significantly predicted guilt decisions. Participants who thought the defendant was an Indian (β = 3.80, *SE* = 0.51, *p <* 0.001), assaulted the victim (β = 5.89, *SE* = 0.59, *p <* 0.001), was recognized by a tribe or the federal government (β = 1.81, *SE* = 0.49, *p* = 0.002), or was categorized racially as an Indian (β = 0.78, *SE* = 0.33, *p* = 0.02) were significantly more likely to say the defendant was guilty than those who said no to any of those questions.

Whether participants thought the defendant had some degree of Indian blood significantly predicted guilt decisions, but not when controlling for individual differences (i.e., feeling thermometer and IMS/EMS). Participants’ external motivations to avoid prejudice was also a significant predictor of guilt (β = 0.17, *SE* = 0.08, *p* = 0.04), such that participants with higher external motivations were significantly less likely to find the defendant guilty. Of note, the first step of the model, with only Indian status determination and assault judgment, accounted for 77% of the variance in participants’ guilt decisions, and adding other indicators of Indian status and measures of individual differences increased the variance accounted for by 2% and 1%, respectively. See Table 5 for full model statistics.

### 7.6. Assault

Given that whether the defendant is an Indian and assaulted the victim were the elements of the crime necessary to find the defendant guilty, we decided also to explore predictors of whether participants thought the defendant committed the assault (for which there are no specific legal elements). To do this, we ran two hierarchical logistic regression models this time predicting mock jurors’ assault decisions (see Table 6 and Table 7). In one (Model 6), we used our manipulated variables (defendant name and photo stereotypicality) as the key predictors, similar to Models 1-4. In the other (Model 7), we included the two factors meant to comprise Indian status (i.e., Indian blood, a recognized member of the tribe) as the key predictors, similar to Model 5. In both models (6 and 7), we added a step that included mock jurors’ Indian status determinations and racial categorization of the defendant.

When predicting assault with our independent variables of defendant name and photo stereotypicality and controlling for individual differences (Model 6), we found no significant predictors until the last step of the model where we added Indian status and Indian race (*r*^2^ = 0.20). In Step 4, the main effect of Indian status was significant (β = 1.93, *SE* = 0.19, *p <* 0.001), the main effect of the defendant’s racial categorization was marginally significant (β = 0.33, *SE* = 0.17, *p* = 0.055), and the conditional effect of defendant photo stereotypicality was significant (β = 0.70, *SE* = 0.33, *p* = 0.03). Mock jurors who thought the defendant met the legal criteria for being Indian, who thought the defendant was racially Indian, and who saw the low (compared to high) stereotypically Indian photo were significantly more likely to say the defendant was guilty of assault. See Table 6 for full model statistics.

**Table 6:** Hierarchical multivariate logistic regression model with name and photo stereotypicality, feeling thermometer, and internal and external motivations to respond without prejudice, indian status, and Indian race predicting assault (0 = No, 1 = Yes).

	Step 1			Step 2			Step 3			Step 4		
*Predictors*	*Odds* *Ratios*	*CI*	*p*	*Odds Ratios*	*CI*	*p*	*Odds* *Ratios*	*CI*	*p*	*Odds* *Ratios*	*CI*	*p*
Intercept	1.29	0.95–1.74	0.10	1.11	0.74–1.66	0.61	0.95	0.38–2.39	0.91	0.40	0.14–1.13	0.08
Low Stereotypical Name	0.90	0.64–1.25	0.53	1.07	0.61–1.88	0.82	1.07	0.60–1.89	0.82	1.03	0.54–1.94	0.94
No Name	1.05	0.74–1.48	0.79	1.41	0.78–2.54	0.26	1.37	0.76–2.48	0.30	1.41	0.73–2.74	0.30
Low Stereotypical Photo	1.26	0.90–1.77	0.18	1.78	1.00–3.18	0.05	1.74	0.98–3.12	0.06	2.01	1.06–3.86	**0.03**
No Photo	0.95	0.68–1.32	0.74	1.06	0.60–1.87	0.83	1.05	0.60–1.85	0.87	1.06	0.56–2.00	0.86
Low Stereo Name × Low Stereo Photo				0.56	0.25–1.25	0.16	0.56	0.24–1.26	0.16	0.66	0.27–1.63	0.37
No Name × Low Stereo Photo				0.64	0.27–1.49	0.30	0.64	0.27–1.52	0.32	0.55	0.21–1.41	0.21
Low Stereo Name × No Photo				1.06	0.47–2.36	0.90	1.05	0.47–2.36	0.90	1.13	0.46–2.80	0.79
No Name × No Photo				0.65	0.28–1.48	0.30	0.66	0.29–1.53	0.34	0.73	0.29–1.84	0.50
Feeling Thermometer							1.10	0.97–1.25	0.13	1.12	0.97–1.29	0.12
IMS							0.95	0.86–1.04	0.28	0.95	0.85–1.05	0.30
EMS							1.02	0.95–1.09	0.59	0.99	0.92–1.07	0.87
Indian										6.90	4.80–10.08	**<0.001**
Racially Indian										1.39	0.99–1.95	0.05
*N*	825			825			825			825		
*R*^2^ Tjur	0.005			0.01			0.01			0.20		

Note. Bolded *p*-values indicate *p* < 0.05. For the name and photo stereotypicality predictors, the high stereotypicality condition is the reference category.

When predicting assault decisions with the factors comprising Indian status (i.e., Indian blood, a recognized member of the tribe) and controlling for individual differences (Model 7), we found significant main effects of the two Indian status factors and Indian status (*r*^2^ = 0.21). Mock jurors who thought the defendant had some degree of Indian blood (β = 0.60, *SE* = 0.22, *p* = 0.006), was recognized as an Indian person by a tribe or the federal government (β = 1.00, *SE* = 0.29, *p* < 0.001), and was therefore an Indian (β = 1.08, *SE* = 0.28, *p* < 0.001) were significantly more likely to say the defendant committed the assault. See Table 7 for full model statistics.

**Table 7:** Hierarchical multivariate logistic regression model with Indian blood, recognized as Indian by tribe or federal government, Indian race, feeling thermometer, internal and external motivations to respond without prejudice, Indian status, and Indian race predicting assault (0 = No, 1 = Yes).

	Step 1			Step 2			Step 3		
*Predictors*	*Odds Ratios*	*CI*	*p*	*Odds Ratios*	*CI*	*p*	*Odds Ratios*	*CI*	*p*
Intercept	0.39	0.26–0.57	<0.001	0.29	0.10–0.79	0.016	0.30	0.11–0.82	0.020
Indian Blood	2.16	1.43–3.31	**<0.001**	2.19	1.44–3.37	**<0.001**	1.82	1.19–2.82	**0.006**
Recognized as Indian	6.96	4.85–10.18	**<0.001**	6.87	4.78–10.06	**<0.001**	2.72	1.53–4.82	**0.001**
Feeling Thermometer				1.09	0.95–1.26	0.23	1.11	0.96–1.29	0.15
IMS				0.97	0.88–1.08	0.58	0.96	0.86–1.06	0.39
EMS				1.02	0.94–1.10	0.62	1.01	0.93–1.09	0.85
Indian							2.93	1.69–5.17	**<0.001**
Racially Indian							1.23	0.88–1.72	0.22
*N*	825			825			825		
*R*^2^ Tjur	0.19			0.19			0.21		

Note. Bolded *p*-values indicate *p* < 0.05. For the categorical predictors, “no” is the reference category.

## 8. Discussion

One aim of our research was to examine whether mock jurors used how stereotypically Indian a defendant’s name and photo were to make their Indian status determinations. Across four models with four different metrics of Indian status, we largely found null effects, contrary to most of our hypotheses, even though we were adequately powered. Of the four questions assessing Indian status, only one is used by the courts: “Did the prosecution prove, beyond a reasonable doubt, that the defendant has some degree of Indian blood?” For this question, none of our variables significantly predicted participants’ responses. When we broke this down and looked at predictors of the two factors that mock jurors are instructed to use to make their Indian status determinations, some degree of Indian blood and recognition by a tribe or federal government, we found internal (for the later) and external (for the former) motivations to respond without prejudice matter, but largely nothing else. As with other studies exploring possible effects of a defendant’s race, we again show that it is important to account for individual differences in prejudice. The one exception to our null findings, however, is that when the defendant’s name and photo were less stereotypically Indian, participants were significantly less likely to say the defendant was recognized by the federal government as an Indian (26%) compared to when the defendant’s photo was less stereotypically Indian and the name was highly stereotypical (42%). Recall that we also explored a third possible factor that jurors may consider in their Indian status determinations: the race of the defendant. Again, we found largely null effects, except when the defendant’s photo was more stereotypically Indian, mock jurors were more likely to say the defendant was of Indian race (40%) compared to when the defendant’s photo was less stereotypically Indian (26.6%).

When considered as a whole, our findings suggest that mock jurors are using some other information to determine whether the defendant is an Indian. Exactly what that is, is an avenue for future research. In our trial materials, multiple different possible indicators of Indian status were kept constant across experimental conditions, but it is possible that one or multiple of these indicators may be crucial to mock jurors’ Indian status determinations. For example, our trial materials stated that the defendant lived on the reservation, attended elementary school at the reservation’s school, and received his primary childhood health care at the reservation’s clinic. We also did not vary the blood quantum of the defendant; in every experimental condition, the defendant was said to be 3/32 Indian, which was less than the tribe’s minimum requirement for membership (1/4). Maybe one of these factors would be a more salient indicator of Indian status to mock jurors than the defendant’s name or appearance. Prior research has found that ancestry and language spoken can be key indicators of whether a person is identified as Indian (Ghoshal, 2024; Schachter et al., 2021), but we did not manipulate these factors in the present study. We can say, however, that how stereotypically Indian a defendant’s name and/or photo are does not have a consistent impact on mock jurors’ Indian status determinations.

The second key aim of our research was to examine predictors of mock jurors’ guilt decisions in trials involving Indian status decisions. Unsurprisingly, we found that mock jurors’ responses to the two questions assessing the elements of the crime necessary to find the defendant guilty (i.e., the defendant is an Indian and assaulted the victim) predicted guilt decisions. Of note, however, is that even when controlling for those elements, as well as internal and external motivations to control prejudice and feelings of warmth towards Indians as a group, we found that participants who believed the defendant’s race was American Indian or Alaskan Native were more likely to find the defendant guilty. This finding may suggest that mock jurors, who have been specifically instructed not to be influenced by the defendant’s race, are, in fact, influenced by the defendant’s race beyond what they are meant to consider in their guilt decisions. Alternatively, the finding of the racial categorization of the defendant as Indian predicting guilt decisions may simply suggest that mock jurors feel race is related to Indian status. When we examined predictors of mock jurors’ assault decisions, instead of guilt, we found that whether participants thought the defendant met the legal criteria for being an Indian was a consistent predictor of assault, with mock jurors who thought the defendant was Indian being more likely to believe the defendant committed the assault. Whether or not the defendant committed the assault, however, should have been independent of mock jurors’ perceptions of whether the defendant was an Indian. Prior research exploring the effects of race and legal decisions has found mixed results, but several studies have found racial bias toward non-White defendants (e.g., Devine & Caughlin, 2014; Maeder & Burdett, 2013; Maeder et al., 2015a; Mitchell et al., 2005), whereas other research finds that racial biases against Black individuals may be less prevalent in legal decision-making research compared to other decisions (Smalarz et al., 2023). Our findings support the prior literature that finds racial biasing effects on mock jurors’ legal decisions, specifically with Indian defendants, but more research is needed to understand why prejudice remains in the legal context for some racial groups but not others. For example, it is possible that the specific instructions in our study for jurors to consider the race of the defendant in their guilt determinations is so starkly different from cases with a Black or other non-White defendant, that it is unsurprising that our research does not replicate recent research on Black defendants (Smalarz et al., 2023).

Our study adds to this complex body of literature in several ways. First, we expand on the impressive research conducted by Maeder and colleagues and examine the effect of possible Indian defendant bias within a U.S. criminal case context. With this, we also designed our study such that we could tease apart the effects of the defendant’s name compared to a photo of the defendant, which prior research on Indian defendants has not done. Although we largely did not find differential effects based on name versus photo, there were some instances in which only the defendant’s photo had an impact on the outcome (i.e., when predicting Indian racial categorization and assault decisions). It is possible that, given the context of the trial (where mock jurors were asked to determine if the defendant was an Indian, and the crime occurred on an Indian reservation), varying the name of the defendant was less impactful. When participants were not given a name or photo of the defendant, just over half still racially classified the defendant as American Indian or Alaskan Native, though only around 30% thought the defendant met the legal criteria for being Indian. These findings add to the prior research on racial categorization (e.g., Chen et al., 2014; Freeman et al., 2011), as well as psycho–legal research examining possible racial biases in legal decision-making.

We also are the first to our knowledge to examine potential biases against Indian defendants using a federal Indian status case, where mock jurors were asked not only to decide guilt but to decide for the courts whether the defendant was an Indian. Our findings show that mock jurors do consider the legal elements necessary to find a defendant guilty (Indian status and commission of the assault), but that mock jurors’ guilt decisions are also influenced by how they racially categorize the defendant and their external motivations to respond without prejudice. Further, when a mock juror determined the defendant met the criteria for Indian status, they were also more likely to think the defendant committed the assault, although the two should be unrelated. Thus, it seems it may be best to bifurcate these two decisions (Indian status and commission of the crime) and use separate juries or to have a judge determine the Indian status of the defendant and a jury determine guilt. This separation would remove some of the additional bias we observed in our study. Given the differing definitions of Indian discussed in our introduction, it may be wise to first settle on one definition of what makes someone an Indian.

## 9. Implications, Limitations, and Future Directions

Our research can help courts refine their jury instructions in Indian status-related trials to better reflect factors the courts do and do not want jurors to consider. Given our results, it may be beneficial for the judge to instruct jurors that the decision of whether the defendant committed the crime is independent of the decision of whether the defendant is an Indian. Our research could also suggest to the U.S. Congress that they take the determination of who is an Indian away from jurors, as we have illustrated through empirical research that having jurors decide Indian status impacts their perceptions of whether the defendant committed the crime. Thus, the U.S. Congress could define “Indian” for purposes of Federal criminal jurisdiction, which would allow jurors in these trials to solely focus on the defendant’s guilt and not their racial background. Although the jury is considered to be a check against government power, allowing “the people” to determine who is guilty of a crime, our research suggests that, in this context, jurors are not serving that function. Further, the findings of this research can help underserved individuals from tribal communities, as our research adds empirical data to an understudied area that directly impacts Indians.

That said, our research is not without limitations. As with most psycho–legal research, our study was limited by an online participant sample, brief mock trial materials, and a lack of jury deliberations. Online samples tend to be more representative of possible juror pools than typical college student samples. Future research should seek to collect participant jurors from community members who were called to jury duty but excused. Our trial materials, although brief (~30 min), were audio recorded by different voice actors, generated by an attorney specializing in Indian law, and based on real cases. We encourage future researchers to create a more extensive video mock trial and have mock jurors deliberate to increase the ecological validity of the research. Our sample is also limited by the lack of control regarding participants’ familiarity with tribal nations, culture, and norms. Future research should assess participants’ knowledge of and exposure to Indian culture to determine whether this may impact their trial decisions. If so, attorneys could use this information to attempt to create a fair and impartial jury, or the U.S. Congress could use the findings to support the idea that jurors should not be used to determine a defendant’s Indian status. Future research should also consider assessing the measures of mock jurors’ prejudice before trial, as opposed to after trial, as done in the current research, to avoid the possibility of the trial impacting responses to those measures.

Another limitation is our manipulation of Indian stereotypicality. Although we pilot-tested the photos and names and matched them on several criteria (perceived age, age, attractiveness, social class, and the likelihood of having a criminal history), it is possible that providing participants with photos of the defendant cued other characteristics/attributes of the defendant that we did not account for. Future researchers could consider blurring the defendant’s face such that skin tone is still evident but other facial features are masked (as done in other research, e.g., Petsko & Bodenhausen, 2019) to avoid additional characteristics being attributed to the defendant. This may not be wise to do when examining Indian defendants, however, as prior research has shown that skin tone is not the most reliable predictor of Indian race (Schachter et al., 2021). Further, given the nature of the trial, our control group simply omitted any racially stereotypical name or photo. This limits the ability to compare our research to previous research on the effects of Indian race on trial decisions, as most prior research uses White defendants as the comparison group.

Last, we observed partial ceiling effects on our Indian blood variable, with just over 80% of participants saying that yes, the defendant had some degree of Indian blood. We believe this is due to our trial materials where we stated the defendant was 3/32 Indian. The trial materials also state that this does not meet the minimum blood quantum required by the tribe to be a member (the requirement was stated to be 1/4 and the attorney clarifies that 3/32 is less than 1/4). Future research should manipulate blood quantum and include as the reference category a defendant who does not have any “Indian blood”. This would allow for a better test of whether a defendant’s blood quantum is an important factor mock jurors consider in their Indian status determinations.

## 10. Conclusions

In sum, our research is the first to explore what factors may impact mock jurors’ Indian status determinations and how these determinations may impact perceptions of the defendant’s guilt. We found that the level of stereotypicality of the defendant’s name and photo did not generally impact Indian status determinations. However, when mock jurors thought the defendant was an Indian, they were more likely to say the defendant committed the assault and was guilty. Although future research is necessary to replicate and extend our results, these findings provide the U.S. Congress and legal actors with preliminary empirical evidence showing the impact having jurors determine who is an Indian for federal criminal jurisdiction has on other important trial decisions (i.e., commission of the crime and guilt).

## Figures and Tables

**Table 3:** Hierarchical multivariate logistic regression model with name and photo stereotypicality, feeling thermometer, and internal and external motivations to respond without prejudice predicting Indian status determinations (0 = Not Indian, 1 = Indian).

	Step 1			Step 2			Step 3		
*Predictors*	*Odds* *Ratios*	*CI*	*p*	*Odds* *Ratios*	*CI*	*p*	*Odds* *Ratios*	*CI*	*p*
Intercept	0.68	0.50–0.92	0.01	0.62	0.41–0.92	0.02	0.54	0.21–1.36	0.19
Low Stereotypical Name	0.96	0.68–1.34	0.80	1.16	0.65–2.07	0.62	1.12	0.63–2.01	0.69
No Name	1.02	0.72–1.44	0.91	1.14	0.63–2.07	0.65	1.09	0.60–1.97	0.79
Low Stereotypical Photo	0.95	0.67–1.33	0.75	1.12	0.63–1.99	0.70	1.09	0.61–1.94	0.77
No Photo	0.94	0.67–1.31	0.70	1.07	0.60–1.91	0.81	1.04	0.58–1.86	0.89
Low Stereo. Name × Low Stereo. Photo				0.59	0.25–1.34	0.21	0.59	0.26–1.36	0.22
No Name × Low Stereo. Photo				1.04	0.45–2.40	0.93	1.10	0.47–2.56	0.83
Low Stereo. Name × No Photo				0.94	0.42–2.13	0.89	0.97	0.43–2.20	0.94
No Name × No Photo				0.68	0.29–1.59	0.38	0.72	0.31–1.67	0.44
Feeling Thermometer							1.03	0.90–1.17	0.70
IMS							0.98	0.89–1.08	0.65
EMS							1.06	0.99–1.13	0.12
*N*	825			825			825		
*R*^2^ Tjur	0.000			0.006			0.01		

Note. For the name and photo stereotypicality predictors, the high stereotypicality condition is the reference category.

**Table 4:** Hierarchical multivariate logistic regression model with name and photo stereotypicality, feeling thermometer, and internal and external motivations to respond without prejudice predicting Indian race determinations (0 = Not Indian, 1 = Indian).

	Step 1			Step 2			Step 3		
*Predictors*	*Odds Ratios*	*CI*	*p*	*Odds Ratios*	*CI*	*p*	*Odds Ratios*	*CI*	*p*
Intercept	2.13	1.56–2.93	**<0.01**	1.85	1.23–2.84	**<0.01**	2.15	0.85–5.51	0.11
Low Stereotypical Name	0.66	0.47–0.92	**0.01**	0.94	0.52–1.70	0.84	0.93	0.51–1.68	0.81
No Name	0.98	0.69–1.39	0.91	1.03	0.56–1.89	0.94	1.02	0.55–1.88	0.96
Low Stereotypical Photo	0.43	0.30–0.61	**<0.001**	0.52	0.29–0.92	**0.03**	0.52	0.29–0.92	**0.02**
No Photo	0.63	0.45–0.88	**0.01**	0.78	0.44–1.40	0.41	0.78	0.44–1.40	0.40
Low Stereo. Name × Low Stereo. Photo				0.52	0.22–1.19	0.12	0.52	0.22–1.20	0.13
No Name × Low Stereo. Photo				1.12	0.48–2.62	0.79	1.14	0.49–2.67	0.76
Low Stereo. Name × No Photo				0.66	0.29–1.50	0.32	0.67	0.29–1.52	0.33
No Name × No Photo				0.79	0.34–1.85	0.59	0.79	0.34–1.85	0.59
Feeling Thermometer							0.96	0.85–1.10	0.57
IMS							1.00	0.91–1.10	0.99
EMS							1.01	0.94–1.09	0.71
*N*	825			825			825		
*R*^2^ Tjur	0.04			0.04			0.04		

Note. Bolded *p*-values indicate *p* < 0.05. For the name and photo stereotypicality predictors, the high stereotypicality condition is the reference category.

**Table 5:** Hierarchical multivariate logistic regression model with Indian status, assault, Indian blood, recognized as Indian by tribe or federal government, Indian race, feeling thermometer, and internal and external motivations to respond without prejudice predicting guilt (0 = Not Guilty, 1 = Guilty).

	Step 1			Step 2			Step 3		
*Predictors*	*Odds* *Ratios*	*CI*	*p*	*Odds* *Ratios*	*CI*	*p*	*Odds* *Ratios*	*CI*	*p*
Intercept	0.00	0.00–0.00	**<0.001**	0.00	0.00–0.00	**<0.01**	0.00	0.00–0.00	**<0.01**
Indian	144.95	68.50–358.79	**<0.001**	44.77	17.79–128.55	**<0.001**	44.51	17.38–130.10	**<0.001**
Defendant Assaulted	273.65	102.31–875.41	**<0.001**	357.27	124.80–1233.75	**<0.001**	362.56	125.49–1267.11	**<0.001**
Indian Blood				0.41	0.19–0.91	**0.03**	0.46	0.20–1.04	0.06
Recognized as Indian				5.84	2.33–15.17	**<0.001**	6.09	2.37–16.39	**<0.001**
Racially Indian				2.01	1.06–3.82	**0.03**	2.18	1.14–4.22	**0.02**
Feeling Thermometer							1.23	0.92–1.65	0.16
IMS							1.03	0.83–1.28	0.80
EMS							1.18	1.01–1.39	**0.04**
*N*	825			825			825		
*R*^2^ Tjur	0.77			0.79			0.80		

Note. Bolded *p*-values indicate *p* < 0.05. For the categorical predictors, “no” is the reference category.

## Data Availability

Study materials are on the project’s OSF page, and the data supporting this research’s findings are available from the corresponding author upon request.

## Note

1 A separate statute covers crimes committed by non-Indians in Indian country when an Indian is the victim. For purposes of these statutes, the U.S. Congress defined the term “Indian country” to include Indian reservations, “dependent Indian communities,” and Indian allotments (Indian Country Statute, 1948). We use the terms “Indian” and “Indian country” to reflect the U.S. Congress’s use of those terms.
